# From Bench to Bedside: Neurofilament Light Chain as a Biomarker in Ischemic Stroke

**DOI:** 10.1007/s11596-026-00200-0

**Published:** 2026-04-28

**Authors:** Khiany Mathias, Anita Dal Bó Tiscoski, Francieli Vuolo, Maria Amélia Cechinel, Naíla Maciel Andrade, Fabricia Petronilho

**Affiliations:** Laboratory of Experimental Neurology, Graduate Program in Health Sciences, Health Sciences Unit, University of Southern Santa Catarina, Criciuma, SC Brazil

**Keywords:** Neurofilament, Ischemic stroke, Neurofilament light chain (NFL), Biomarker

## Abstract

Neurofilaments (NFs) are protein structures that form part of the neuronal cytoskeleton, providing structural support and facilitating axonal transport. Neurofilament light chain (NFL) has emerged as a promising biomarker for various neurological conditions, as it is released into the bloodstream following neuronal damage. The ability of NFL to differentiate between types of injury and enhance the accuracy of clinical prediction models makes it an asset in both clinical practice and research. Studies suggest that NFL can be employed to diagnose and monitor disease progression and aid in the prognosis of various neurological conditions, including ischemic stroke (IS). In IS, NFL serves as a predictor of severity, functional recovery, and the risk of complications. This review therefore synthesizes current preclinical and clinical studies on the use of NFL as a biomarker for prognosis, monitoring, and treatment in IS.

## Introduction

Ischemic stroke (IS) is a leading cause of death and disability worldwide, resulting in neuronal loss and impairment of neurological functions. Early and accurate detection of brain injury is crucial for therapeutic interventions and prognosis [1–3]. In this context, the neurofilament light chain (NFL) has emerged as a promising biomarker of neuronal damage in IS [4].

Neurofilaments (NFs) are structural proteins that make up the cytoskeleton of neurons and are released into the cerebrospinal fluid (CSF) and bloodstream after neuronal injury because of neuronal death, axonal dysfunction, and disruption of the blood‒brain barrier (BBB) [1, 2]. In particular, NFL has been widely investigated as a sensitive blood biomarker of neuronal injury for detecting neuronal damage in various neurological conditions, such as amyotrophic lateral sclerosis [5], cerebral malaria [6], neuroaxonal injury and peripheral neuropathy [7], chronic traumatic brain injury (TBI) [8], spinal cord infarction from acute myelitis [9], small vessel disease [10], Alzheimer’s disease [11], and IS [12, 13]. With respect to IS, a recent study revealed that NFL levels increase in the blood soon after an IS, which is correlated with the severity of the injury, infarct size, and functional prognosis of patients [14].

In this review, we explore preclinical and clinical studies on NFLs as biomarkers in the context of IS. We discuss the potential of NFLs not only to increase diagnostic accuracy but also to predict functional recovery, assess the risk of complications, and monitor disease progression. This approach highlights the promise of NFLs in enabling more precise and individualized management of patients affected by IS.

Although previous reviews have addressed the role of NFL in neurological diseases and stroke, most have focused primarily on either clinical prognostic value or biomarker performance in isolated contexts. The present review provides an updated and integrated bench-to-bedside perspective, combining preclinical mechanistic evidence with contemporary clinical findings. Additionally, we discuss methodological considerations, temporal dynamics, and translational challenges that may influence the implementation of NFL in routine stroke care.

## NFs: Structure and Composition

NFs are neuron-specific intermediate filaments that play a critical role in maintaining neuronal structure and function. They are composed of four main subunits in the central nervous system, namely, the NFL, neurofilament medium chain (NFM), and neurofilament heavy chain (NFH), along with α-internexin, while peripherin replaces α-internexin in the peripheral nervous system [15]. These subunits assemble into 10 nm diameter filaments, with NFL and α-internexin/peripherin forming the filament core and NFM and NFH extending outward as side arms rich in phosphorylation sites [15].

Each NF subunit comprises a conserved central α-helical rod domain, a head domain at the amino-terminal end, and a variable carboxy-terminal tail. The long C-terminal tails of NFMs and NFHs contain multiple lysine–serine–proline (KSP) repeats that serve as major phosphorylation sites, facilitating interactions with other cytoskeletal proteins and contributing to filament stability [15, 16]. In contrast, NFL, α-internexin, and peripherin have shorter tails and lack KSP motifs [16].

NF assembly begins with the formation of coiled-coil dimers, which organize into tetramers and then unit-length filaments (ULFs). These ULFs subsequently elongate and compact to form mature NFs [15]. Computational models suggest that side arms, particularly from the NFH, extend radially from the filament core, forming a dense, cross-linked network that supports axonal structure with minimal metabolic cost [17, 18].

The expression of NFs is developmentally regulated. NFL and NFM are expressed earlier, giving rise to immature, more plastic filaments. NFH is incorporated later during axonal maturation and synaptogenesis, increasing filament stability [19]. The relative abundance of NFH increases throughout development, with variations observed across species, cell types, and brain regions. Its presence confers resistance to depolymerizing agents such as colchicine, underscoring its role in cytoskeletal resilience [19].

The phosphorylation of NFM and NFH is associated with key processes such as axonal radial growth and myelination, suggesting that these proteins play a role not only in mechanical support but also in modulating axonal transport and organelle dynamics [19]. Moreover, the heterogeneous distribution of NFs across neuronal subtypes indicates that they may also contribute to the functional specialization of neurons [18].

## NFL as a Biomarker of Brain Damage

NFL has emerged as a promising biomarker for various neurological conditions, revealing important information about brain health [20]. Its presence in the blood indicates neuronal damage, and recent studies have explored its potential to detect and monitor different brain diseases. Research has indicated that elevated serum NFL (sNFL) levels may be related to aging and cognitive decline [21], Parkinson’s disease [22], Huntington’s disease [23], multiple sclerosis [24], Alzheimer’s disease, encephalitis [25], TBI [26], cancer with neurological complications [27] and even the influence of diet on brain health [28].

Although studies describe ‘high’ or ‘low’ sNFL levels, the definitions of these thresholds vary. In clinical studies, sNFL levels in healthy adults typically range from 5 to 20 pg/mL and increase gradually with age. After IS, concentrations above 50–100 pg/mL have been reported within days of the event, with peaks reaching more than 200 pg/mL in severe cases [22, 29]. In terms of detection, in neurodegenerative conditions, sNFL has comparable diagnostic performance to that of CSF NFL, especially in younger people [30, 31].

With respect to the correlation between the aging process and neurological impairment, higher sNFL levels are associated with lower brain volume, more white matter lesions, and a faster decline in thinking skills over time, especially in people older than 60 years [20]. A general population study confirmed the relationship between elevated sNFL levels and poorer cognitive performance, especially in language, praxis, and memory [21]. Furthermore, these elevated levels, combined with advanced age, were associated with markers of changes in brain regions, such as atrophy in the medial temporal lobe and white matter lesions [21].

In Parkinson’s disease, high sNFL levels are associated with faster disease progression and steeper cognitive decline over time [22]. Furthermore, sNFL is the best predictor of disease progression, outperforming other biomarkers, such as p-tau181 and glial fibrillary acidic protein (GFAP), and is correlated with the severity of motor and nonmotor symptoms [29]. In a study with mice with the A53T mutation for Parkinson’s disease, the increase in NFL in the CSF and plasma confirmed neurodegeneration and the presence of symptoms such as muscle weakness, motor problems, α-synuclein accumulation in the brain, abnormal fibers and neuroinflammation [31].

In patients with Alzheimer’s disease, an increase in NFL can be detected years before the onset of dementia symptoms, contributing to early diagnosis [25]. Studies have revealed that genes that influence blood NFL levels have been identified, some of which are linked to neurodegeneration and Alzheimer’s disease [32]. Both NFL and GFAP are related to worsening neuropsychiatric symptoms in people without dementia [33].

Additionally, in people without dementia, higher blood NFL levels predict worse cognition, reduced hippocampal volume, and increased white matter lesions [34]. The presence of amyloid plaques and hypertension intensified the effects of NFL on these brain changes [34].

In individuals diagnosed with amyotrophic lateral sclerosis (ALS), there is a notable increase in blood levels of NFL [35]. Of particular interest, the investigation also revealed a correlation between a genetic predisposition for ALS (*UNC13A*) and the levels of TDP-43 in the bloodstream; however, no such correlation was established with NFL levels [35]. Additionally, regarding neurodegenerative conditions in murine models of Huntington’s disease, a decrease in NFL levels in both CSF and blood was documented in mice that underwent treatment aimed at mitigating disease-inducing protein expression [23].

In addition to neurodegenerative diseases, elevated blood NFL levels have been observed in various acute and immune-mediated neurological disorders, reflecting neuroaxonal injury. In patients with cancer and neurological complications, high NFL concentrations are associated with greater disease severity and an increased risk of death [27]. Similarly, in infectious encephalitis, such as that caused by HIV, HSV, HZV, and SARS-CoV-2, increased NFL reflects both disease severity and neurological impairment. In autoimmune encephalitis, increased NFL levels parallel neuroaxonal damage and serve as a biomarker to monitor recovery [25]. In the case of multiple sclerosis, NFL is a well-established biomarker for monitoring disease activity and disability progression [24, 25]. It can detect disease activity even before the onset of symptoms or brain lesions, aiding in treatment decisions [24].

With respect to moderate–severe TBI, a study confirmed the presence of persistent brain injury in people who had been diagnosed with TBI more than 10 years ago through increased blood NFL [26]. NFL is correlated with white matter lesions and memory impairment, demonstrating its usefulness for monitoring brain health after TBI [26]. In cardiac surgeries, NFL levels are particularly elevated, especially in aged patients who develop delirium. NFL may therefore be useful for identifying patients at risk of neurological complications after surgery, guiding preventive measures and optimizing treatment [36].

The composition of fatty acids in the blood can also influence NFL levels [28]. High levels of certain saturated fats were related to lower NFL, whereas high levels of certain unsaturated fats were associated with higher NFL. These findings may open avenues for investigating the role of diet in brain health [28].

NFL has emerged as a promising tool for assessing and monitoring different brain diseases and guiding diagnosis, treatment, and prognosis. Research on NFL continues to expand the possibilities for the application of this biomarker, contributing to the advancement of neurology and the improvement of brain health. In this sense, recent studies have investigated the potential of NFL as a biomarker for IS, evaluating its ability to predict the severity of neurological damage, assist in prognosis, and monitor patient recovery.

## NFL In IS: Preclinical Evidence

Preclinical studies have played a pivotal role in establishing the NFL as a reliable biomarker of neuronal injury in the context of IS. The molecular properties described above, including NF phosphorylation dynamics, cytoskeletal remodeling, and release mechanisms following axonal injury, provide the biological basis for the detection of circulating NFL after IS. Disruption of axonal integrity and BBB permeability facilitates the release of NFL into extracellular fluids, ultimately enabling its measurement in plasma and serum. These mechanistic aspects strengthen the rationale for its application as a translational biomarker in IS. In animal models, particularly in aged rodents subjected to distal middle cerebral artery occlusion (MCAO), the NFL has been extensively investigated in both plasma and brain tissue, offering crucial insights into its pathophysiological relevance and translational potential (Table 1).

**Table 1:** NFL as a biomarker and prognostic marker in experimental stroke

Experimental model	Tissue analyzed (assay used)	Participants	Association with disease	References
Distal MCAO model + hypoxia (DH)	Plasma (Quanterix Simoa^®^ NF-Light v2 Advantage assay)	C57BL/6 mice	Biomarker of neuroaxonal damage	Becktel et al. [37]
Mice: Permanent MCAO on the right side, permanent thromboembolic MCAO on the right side (EMCAO) Sheep: Surgically induced permanent MCAO	Immunohistochemistry (rabbit-anti-neurofilament L; biotinylated rabbit-anti-neurofilament L IgG; guinea pig-anti-neurofilament M; guinea pig-anti-neurofilament H; biotinylated mouse-anti-neurofilament H IgG [clone NE14]) Western blot (mouse-anti-neurofilament L [clone DA2]; rabbit-anti-neurofilament L)	C57BL/6 male adult mice (25 g); Wistar male adult rats (300 g) Sheep	NFL increases in areas affected by stroke, including in the penumbral region NFL as an important marker for identifying areas of the brain affected by stroke	Mages et al. [38]
Middle cerebral artery occlusion (EMCAO)	Tissues and sections of the forebrain, from rats and mice with experimentally induced stroke (rabbit-anti-NF-L [1:100]) Human autopsy brain tissue (Rabbit-anti-NF-L [1:100])	Wild-type Sv129/B6 mice (30 g); Wistar rats (300 g) A 63-year-old man with an infarct in the territory of the right middle cerebral artery	Positive regulation of NFL after experimental stroke Strong positive regulation of NFL in human autopsy tissue from a stroke patient	Härtig et al. [39]

In plasma, compared with those in control groups, NFL levels were significantly elevated and sustained for at least seven weeks following MCAO, indicating prolonged neurodegenerative processes [37]. Moreover, a strong positive correlation was observed between NFL concentrations measured 24 h post-occlusion and infarct volume, suggesting that early plasma NFL (pNFL) levels may reflect the severity of acute cerebral injury [37].

At the tissue level, increased NFL expression has been reported, particularly in the ischemic penumbra, which is the region surrounding the infarct core that retains the potential for functional recovery [38]. Cerebral ischemia induces a cascade of molecular events that disrupt neuronal homeostasis, including cytoskeletal reorganization. This remodeling is characterized by the degradation or loss of structural proteins such as microtubule-associated protein 2 (MAP2) and tau, along with increased accumulation of NFL [39–42].

Interestingly, MAP2 reduction and NFL accumulation tend to occur in complementary brain regions, suggesting region-specific vulnerability and distinct responses to injury [39]. While the decrease in MAP2 is likely a result of protein degradation, the increase in NFL may reflect both the release of its degradation products and active reorganization of the cytoskeleton [40]. This dynamic provides valuable insight into the spatial and temporal evolution of ischemic damage.

Given these properties, NFL has attracted attention not only as a marker of acute injury but also as a tool for tracking postischemic progression and assessing therapeutic interventions [41]. In this context, novel strategies such as pulsed electromagnetic fields (PEMFs) have been investigated. PEMF, applied at specific frequencies, promotes stem cell differentiation into neurons and modulates the expression of NFL and other cytoskeletal proteins [42]. Additionally, PEMF may increase neurogenesis and reduce neuroinflammation, supporting recovery after ischemic injury [42].

Taken together, these findings reinforce the importance of targeting the neuronal cytoskeleton, particularly NFL, in the development of innovative diagnostic and therapeutic approaches for IS (Table 1).

## NFL in IS: Clinical Evidence

NFL has emerged as a promising biomarker for neuronal injury in IS, offering insights into both acute damage and long-term prognosis. Following IS, serum and plasma NFL levels increase significantly, typically peaking around the seventh day and remaining elevated for weeks to months, reflecting sustained neurodegeneration and limited neuronal repair [43, 44]. These levels are consistently associated with infarct volume and stroke severity in the acute and subacute phases [13, 14], although these correlations may weaken after adjustment for confounding factors such as age and initial neurological deficits [45].

Even among patients with similar clinical recovery, those with cortical lesions exhibit higher NFL levels than those with subcortical strokes do, indicating greater underlying structural damage and cortical vulnerability [46]. In small vessel disease, elevated NFL levels are observed both acutely and at six months, and these levels correlate with white matter hyperintensity (WMH) burden, even in the absence of overt atrophy [47]. Although sNFL in the early post-stroke period is more strongly associated with infarct volume than with WMH progression, a longitudinal study revealed that after seven years, NFL levels moderately correlated with increased WMH burden over time [13], suggesting long-term predictive value (Table 2).

**Table 2:** NFL as a biomarker and prognosticator in clinical stroke

Study design	Tissue analyzed (assay used)	Participants	Association with disease	Reference
Prospective cohort study	Plasma (Quanterix Simoa^®^ on the SR-X biomarker detection system)	3077 patients with atrial fibrillation and increased risk of stroke	Elevated NFL levels associated with increased risk of stroke/TIA, and increased risk of cardiovascular death	Aulin et al. [50]
Prospective cohort study	Blood (Quanterix Simoa^®^)	316 IS patients (65% men, mean age 53 years) and at 7-year follow-up (n = 187)	Correlation between sNFL and infarct volume in the acute and subacute phase of stroke Correlation between sNFL and WMH volume in the acute phase and at 7 years of follow-up sNFL as a biomarker for infarct size in the acute and subacute phase after IS	Holmegaard et al [13]
Prospective cohort study	Blood (ELISA)	319 acute IS patients	High NFL after IS associated with increased risk of complications and worse functional recovery at 3 months NFL + NIHSS + white matter lesions associated with better prediction of complications and recovery	Xu et al. [68]
Longitudinal prospective cohort study	Plasma (Quanterix Simoa^®^ on the SR-X analyzer)	13 patients (4 women, 74.7 ± 8.8 years) who suffered a stroke in the previous month and were hospitalized for 2 months of rehabilitation	NFL correlates with the extent of the lesion	Lanzone et al. [46]
Single-center prospective cohort study	Serum (BioTechne Ella automated ELISA)	213 stroke patients (47% women, 76 ± 12 years)	Elevated NFL levels in serum after acute IS were associated with worse functional status at 3 months NFL is an independent predictor of unfavorable recovery	Vollmuth et al. [48]
Cohort study	Serum (BioTechne Ella automated ELISA, Quanterix Simoa^®^ GFAP* on an HD-X platform)	30 adult patients with acute IS, assessed one day after admission, moderate to severe due to MCAO	Higher NFL in blood soon after stroke correlated with larger brain lesions and worse initial neurological recovery Patients with elevated NFL had worse functional recovery after 3 months of stroke	Barba et al. [12]
Longitudinal prospective cohort study	Serum (Quanterix Simoa^®^ NFL Advantage Kits, #103,400)	36 stroke patients within 24 h of symptom onset, followed up at 7, 30, and 90 days	Elevated sNFL levels after stroke were associated with greater severity and worse functional recovery	Ferrari et al. [69]
Prospective cohort study	Serum (Quanterix Simoa^®^)	152 minor acute IS patients within 24 h of symptom onset	Higher sNFL levels in patients with multiple infarcts compared with healthy controls Patients with MIS who develop encephalopathy and neurological deterioration have even higher sNFL levels	Li et al. [49]
Population-based cohort study	Blood (Quanterix Simoa^®^ HD-X bead-based assay and the Neurology 4-Plex)	3,603 person-years of follow up, 133 individuals developed incident stroke, including ischemic and hemorrhagic	Association of NFL with the risk of stroke Association of NFL with the presence of cerebral infarcts	Dhana et al. [70]
Prospective cohort study	Serum (Quanterix Simoa^®^ NFL Light assay)	4,016 patients submitted to cardiac exams for suspected CAD	Risk of stroke/TIA associated with sNFL Prognostic value of sNFL for cardiovascular and cerebrovascular events	Amrein et al. [52]
Single-center prospective cohort study	Serum (Quanterix Simoa^®^)	51 patients with moderate to severe IS	Higher NFL levels were also associated with an increased risk of hemorrhagic transformation	Rattanawong et al. [71]
Single-center prospective cohort study	Serum (Quanterix Simoa^®^ NFL-light Advantage Kits on an HD-X analyzer)	15 first-ever acute IS patients and 8 healthy controls	Correlation between NFL and infarct volume Relationship between NFL and stroke severity or functional recovery	Ahn et al. [14]
Prospective cohort study	Plasma (Simoa)	264 patients, of whom 101 were clinically diagnosed with post-stroke cognitive impairment (PSCI)	Importance of pNFL as a predictor of PSCI in posterior circulation stroke Relationship between pNFL and cognitive impairment in anterior circulation stroke	Jiang et al. [57]
Prospective cohort study	Plasma (immunoassay [ECL] on the Meso Scale Discovery [MSD] platform)	60 acute IS patients	Higher pNFL levels correlated with greater stroke severity, worse neurological recovery, and less ability to perform daily activities	Wu et al. [43]
Prospective cohort study	Serum (Quanterix Simoa^®^ assay kit, Neurology 4-Plex A/Simoa HD-1)	110 acute IS patients	Correlation between sNFL and NIHSS scores Association between sNFL and mRS scores at 6 months after stroke	Zhou et al. [63]
Prospective cohort study	Plasma (Quanterix Simoa^®^ NFL-light Advantage Kits)	4,661 participants without stroke from the population-based Rotterdam Study	Increase in NFL showed a strong association with the risk of hemorrhagic stroke	Heshmatollah et al. [67]
Prospective cohort study	Serum (Simoa)	304 acute IS patients	NFL was able to predict the occurrence of severe depression, anxiety, and insomnia	Wang et al. [54]
Prospective cohort study	Plasma (Quanterix Simoa^®^ Cytokine 4-Plex on the Simoa HD-1 Analyzer)	54 acute IS patients	Increased NFL levels associated with increased stroke risk Correlation between NFL levels and stroke	Correia et al. [64]
Prospective cohort study	Plasma (Neurology 4-Plex/Quanterix Simoa^®^)	60 patients who underwent endovascular thrombectomy for acute IS	Higher NFL levels at all three time points (before, immediately after, and 24 h after EVT) were independent predictors of death or disability at 90 days	Chen et al. [62]
Longitudinal prospective cohort study	Serum (Simoa)	304 patients with subjective PSCI	NFL in blood proved to be an important predictor of recovery	Wang et al. [55]
Single-center prospective cohort study	Plasma (Quanterix Simoa^®^)	1,694 patients with PSCI	Elevated NFL levels in patients with PSCI	Wang et al. [56]
Prospective cohort study	Serum (Quanterix Simoa^®^ NFL Assay Kits, #103400)	144 acute IS patients and 30 patients without stroke	Correlation between NFL and infarct volume Association between NFL and cognitive function Predictive value of NFL for cognitive outcome	Peng et al. [58]
Retrospective cohort study	Plasma (Quanterix Simoa^®^ NFL-Light Digital Immunoassay, #103186)	393 individuals (314 patients with acute cerebral infarction, aSAH or intracerebral hemorrhage and 79 control individuals)	Association between NFL and neurological, functional and cognitive status Predictive value of NFL for functional outcome at 3 months Predictive value of NFL for survival	Gendron et al. [65]
Prospective cohort study	Plasma (Quanterix Simoa^®^ HD-1 Analyzer)	2 post-mortem cases of human IS, representing infarcts aged 3 to > 7 days	Correlation between NF-L and stroke severity Correlation between NF-L and functional outcome	Nielsen et al. [72]
Single-center prospective cohort study	Serum (Quanterix Simoa^®^ HD-1 Analyzer)	454 first-ever acute IS patients	NFL is a sensitive indicator of central nervous system axonal injury sNFL is a novel and independent predictor of 3-month depression in IS High sNFL levels are correlated with higher NIHSS score, larger infarct size, and worse functional outcome	Zhao et al. [53]
Cohort study	Serum (Quanterix Simoa^®^ HD-1 using the NFL-Light Advantage Kits)	211 patients with median clinical severity on NIHSS score	Correlation between sNFL and stroke severity Association between sNFL and 90-day recovery Association between sNFL and long-term cardiovascular events	Uphaus et al. [66]
Cohort study	Serum (Quanterix Simoa^®^)	595 acute IS patients with serum samples	Higher sNFL levels were associated with worse neurological and functional recovery	Pedersen et al. [44]
Single-center prospective cohort study	Serum (Quanterix Simoa^®^, digital ELISA)	136 patients admitted for acute IS or TIA	sNFL concentration correlated with stroke severity, infarct size, and functional recovery after 3 months	Onatsu et al. [73]
Multicenter prospective cohort study	Plasma (NF-light^®^ assay, Simoa HD-1 Analyzer, Quanterix)	134 acute IS patients	NFL elevation correlated with cerebral infarct size and the occurrence of new ischemic lesions	Tiedt et al. [74]
Prospective cohort study	Serum and CSF (UmanDiagnostics kit ELISA, and Simoa)	30 acute IS patients	The highest concentration of NFs appears 3 weeks after IS NFs correlate with the performance of activities of daily living after stroke	Calderón et al. [60]
Prospective cohort study	Serum (ELISA, electrochemiluminescence immunoassay, and Simoa)	504 patients with acute IS and 111 patients with TIA	sNFL levels correlated with stroke severity at admission but not with infarct size	Marchis et al. [45]
Prospective cohort study	Serum (Simoa)	95 patients with recent small subcortical infarcts confirmed by MRI	NFL levels correlated with time since symptom onset and stroke severity Higher NFL levels were also associated with markers of small vessel disease	Gattringer et al. [61]
Proof-of-concept observational study	Serum (electrochemiluminescence immunoassay)	49 cervical artery dissection patients	Elevated sNFL levels indicated greater stroke severity and worse prognosis at 3 months	Traenka et al. [75]
Prospective cohort study	Serum (EnCor Biotechnology, Inc. ELISA kit of pNfH pre-coated)	54 individuals with acute IS	Blood levels of pNfH increase after IS, correlating with stroke severity and functional recovery at 6 months	Singh et al. [76]

In addition to its diagnostic relevance, NFL has shown consistent potential as a prognostic biomarker. Higher levels are associated with worse functional outcomes and greater disability at 3 to 6 months post-stroke, including in patients with large infarcts where sNFL increased between admission and the following day, suggesting delayed yet progressive release following neuronal damage [12, 45, 48]. Furthermore, patients with multiple infarcts or those who developed post-stroke encephalopathy and neurological deterioration have even higher sNFL levels, despite having similar initial stroke severity [49], supporting its value in detecting hidden or evolving damage.

NFL also offers predictive insights into primary and secondary prevention. Elevated baseline sNFL levels have been associated with an increased risk of IS or transient ischemic attack (TIA), particularly in individuals with no previous history of cerebrovascular disease [50]. In diabetic populations, NFL levels were strongly predictive of stroke risk over a seven-year period, with individuals in the highest range presenting up to an 11-fold increased likelihood of IS [51]. While NFL is not specific for coronary artery disease (CAD), its levels predict future cerebrovascular events more accurately than traditional CAD biomarkers do [52]. Additionally, in patients with atrial fibrillation, NFL was associated with both cardiovascular and all-cause mortality, further reinforcing its potential as a systemic health marker [50].

NFL has also demonstrated value in predicting cognitive and neuropsychiatric outcomes after IS. High serum levels have been associated with a high risk of post-stroke depression, anxiety, and insomnia [53, 54], as well as long-term cognitive impairment [55, 56]. These associations remained significant even after adjustment for confounders such as age, lesion volume, and baseline neurological status. In patients with posterior circulation strokes, elevated plasma NFL in the acute phase predicted cognitive decline after 90 days [57], whereas in rehabilitation settings, NFL levels outperformed traditional scales in predicting cognitive recovery at discharge [58].

The timing of NFL measurement plays a critical role in its clinical utility. Early collection (within 24 h) may lead to the underestimation of injury, whereas samples collected between days 3 and 7 have stronger prognostic associations [4, 59]. NFL levels measured on day 7 showed superior predictive value for functional recovery compared with baseline levels [43]. In cerebrospinal fluid (CSF) and blood, NFL levels increase in parallel, peaking in the third week and slowly declining thereafter [60]. Some patients maintain elevated levels for up to 15 months post-stroke, particularly those who develop new asymptomatic lesions [61], highlighting the progressive and silent nature of cerebrovascular injury. In addition to biological and temporal variability, methodological heterogeneity across studies represents an important technical limitation in the interpretation of circulating NFL levels. Different analytical platforms have been used to quantify NFL, including conventional enzyme-linked immunosorbent assay (ELISA) and ultrasensitive technologies such as single-molecule arrays (SIMOA). SIMOA allows the detection of substantially lower NFL concentrations in serum and plasma because of its higher analytical sensitivity, whereas ELISA-based assays generally have higher detection thresholds. Consequently, absolute NFL values and reported cutoff levels may not be directly comparable between studies using different platforms. The lack of international assay standardization and harmonized reference ranges further contributes to interstudy variability and currently limits the establishment of universally accepted diagnostic and prognostic thresholds. These methodological aspects should be carefully considered when interpreting NFL data and when translating research findings into clinical practice.

With respect to the response of NFL levels to acute therapies, in patients who underwent endovascular thrombectomy (EVT), elevated NFL levels before and shortly after the procedure were associated with a greater risk of disability and death at 90 days, regardless of the degree of recanalization achieved [62]. Importantly, the predictive value of NFL was more pronounced in patients who did not receive revascularization treatment [63]. Furthermore, the rate of increase in NFL levels may have additional prognostic value, even if not always statistically significant [64].

Despite its promise, NFL is not disease specific. Its levels can increase in various neurological and systemic conditions, including neurodegenerative diseases, TBI, and infections. Thus, the interpretation of NFL must be contextualized with imaging findings, clinical scales (e.g., National Institutes of Health Stroke Scale [NIHSS], modified Rankin Scale [mRS]), and other biomarkers to improve specificity [59]. Multimodal approaches, including dynamic profiling and biomarker panels, are likely to enhance the clinical applicability of NFL.

This broader perspective has been reinforced by meta-analyses, which confirm that elevated NFL levels are consistently correlated with stroke severity, poor functional outcomes, long-term dependence, and increased mortality [4, 59, 65]. Compared with traditional biomarkers such as N-terminal pro-B-type natriuretic peptide (NT-proBNP), atrial natriuretic peptide (ANP), and fatty acid-binding protein (FABP), NFL has demonstrated superior prognostic accuracy [66]. Moreover, NFL measured several months post-stroke can predict recurrent cerebrovascular events and long-term outcomes with notable sensitivity.

Finally, longitudinal studies have identified NFL as a reliable predictor of stroke risk in high-risk populations. A cohort followed for more than 10 years revealed that elevated CSF NFL and total tau were associated with increased stroke risk, especially among individuals with the APOE ε4 genotype [67]. Together, these findings support the role of NFL not only as a marker of acute neuronal injury but also as a biomarker with long-term predictive power that is useful for guiding rehabilitation, identifying patients at high risk, and supporting preventive strategies for cerebrovascular disease.

## Conclusion

NFL has emerged as a sensitive and reliable biomarker of neuronal injury with broad applicability in neurological disorders, including IS. This review synthesizes robust preclinical and clinical evidence demonstrating that NFL levels in blood and cerebrospinal fluid reflect the extent of neuroaxonal damage, correlate with infarct volume and stroke severity, and predict both functional recovery and long-term cognitive outcomes. Preclinical models have helped elucidate the molecular dynamics of NFL expression following cerebral ischemia, while clinical studies have validated its prognostic relevance in diverse patient populations and across different stroke subtypes.

Beyond its value in acute assessment, NFL has shown promise in identifying patients at risk for complications such as neurological deterioration, depression, and cognitive decline. Its ability to track subclinical damage, enhance risk stratification, and complement neuroimaging and clinical scales reinforces its utility in both personalized care and clinical research. However, while NFL is a powerful tool, its lack of disease specificity and variability in measurement timing underscore the importance of contextual interpretation and standardization.

Taken together, the integration of the NFL into multimodal diagnostic and prognostic frameworks may represent a significant advance in stroke management. Future studies should aim to refine reference thresholds, assess cost-effectiveness, and explore its utility in guiding therapeutic decisions. Ultimately, the use of NFL as a biomarker in IS has the potential to improve early diagnosis, optimize patient monitoring, and personalize rehabilitation strategies, contributing to better clinical outcomes.

Future research should prioritize the establishment of standardized sampling protocols, including optimal timing of blood collection and harmonization of analytical platforms. Large, multicenter prospective studies are needed to define clinically actionable NFL thresholds adjusted for age and comorbidities. Additionally, integrating NFL into multimodal prognostic algorithms combining neuroimaging markers, clinical scales (e.g., NIHSS, mRS), and other blood biomarkers may increase its predictive accuracy. Finally, cost-effectiveness analyses and implementation studies will be essential for determining the feasibility of incorporating NFL into routine stroke care pathways.

## Data Availability

Not applicable.
